# Closing Editorial for the Special Issue: “Advanced Treatment of Schizophrenia”

**DOI:** 10.3390/biomedicines13112752

**Published:** 2025-11-11

**Authors:** Felix-Martin Werner, Rafael Coveñas

**Affiliations:** 1 Grone Gesundheitsakademie Weimar, Otto-Schott-Straße 2, 99427 Weimar, Germany; 2 Laboratory of Neuroanatomy of the Peptidergic Systems, Institute of Neuroscience of Castilla y León (INCYL), University of Salamanca, 37007 Salamanca, Spain; covenas@usal.es; 3 Group GIR USAL, BMD (Bases Moleculares del Desarrollo), University of Salamanca, 37007 Salamanca, Spain

Schizophrenia, a chronic disabling disease, is a complex psychiatric disorder with a global prevalence of approximately 1%. In this disease, positive schizophrenia symptoms—for example, paranoia, hallucinations, and above all, acoustic hallucinations and illusions—occur. These positive symptoms can be effectively ameliorated by the administration of first- and second-generation antipsychotic drugs [1]. Negative schizophrenia symptoms are social withdrawal, reduced communication, and depression. Currently available antipsychotic drugs can only partially alleviate these negative symptoms [2,3,4,5,6,7,8,9,10,11,12,13,14]. Moreover, cognitive symptoms of schizophrenia, for example, a reduced concentration, cannot be treated at all using current pharmacotherapy [14]. Therefore, investigations into new or improved antipsychotic drugs, improving the cognitive behavior of patients, and reducing hospitalizations are of a great importance in schizophrenia. Moreover, research on the treatment of therapy-resistant forms of schizophrenia must not be neglected. In this Editorial, we outline the main findings reported in the articles published in this Special Issue, which aims to increase basic knowledge of schizophrenia and suggest promising treatments of the disease, such as the following: risperidone ISM^®^ [15], triglycerides and remnant cholesterol levels and systemic inflammation [16], cytokines and electroconvulsive therapy [17], cognition and computer-assisted cognitive remediation therapy [18], schizophrenia and electroconvulsive therapy [19], schizophrenia and neurological soft signs [20], and negative symptoms and pharmacological intervention [21].

One of the often-prescribed antipsychotic drugs for schizophrenia is risperidone. A group of Spanish medical doctors, under the leadership of Francisco José Toja-Camba, examined the administration of risperidone ISM^®^ in patients with schizophrenia and studied some clinical parameters. Risperidone ISM^®^ is a long-acting injectable formulation that has been approved for monthly administration [15]. A cohort of forty-four patients suffering from schizophrenia received risperidone ISM^®^ in a dosage of 75 or 100 mg; pharmacokinetic parameters (AUC, Tmax, Cmin, Cmax) were examined in a real-world clinical setting, and injection sites (deltoid or gluteal) were also studied. The long-injectable form of risperidone reached therapeutic plasma levels within hours after administration and remained constant during twenty-eight-day interval [15]. The authors stated that significant interindividual variability was observed and that the higher systemic exposure was found when risperidone ISM^®^ was administered in the deltoid [15]. Thus, risperidone ISM^®^ achieved sustained and rapid therapeutic plasma levels, meaning that risperidone ISM^®^ is a useful tool to treat patients with schizophrenia, although the high interindividual variability reported by the authors must be studied in-depth. Systemic inflammation and hypertriglyceridemia occur in patients with schizophrenia, increasing the risk for cardiovascular diseases [16]. A group of Australian researchers under the leadership of Jeffrey Wang, examined cholesterol and triglyceride levels and inflammation markers in a cohort of patients with schizophrenia (147 patients received antipsychotic drugs) and in a group of healthy subjects (56 individuals) [16]. Most patients were administered with clozapine, whereas others received quetiapine, paliperidone, risperidone, aripiprazole, clopixol, or amisulpride. The authors found that remnant cholesterol and triglyceride levels increased in schizophrenic patients; however, the inflammation markers did not correlate with the severeness of this increase. The level of interleukin (IL)-10 augmented in patients with schizophrenia suggesting an anti-inflammatory action [16]. The findings of the study show that patients with schizophrenia have independent risk factors for secondary diseases from arteriosclerosis, for example, myocardial infarct or coronary heart disease [16]. In the third research article, a group of Polish psychiatrists under the leadership of Anna Maria Szota examined changes in the levels of cytokines (IL-6, IL-12, IL-5, IL-10, transforming growth factor (TGF)-β1) in eight patients with therapy-resistant schizophrenia (TRS) who underwent an electroconvulsive therapy [17]. Because TRS is considered a neuro-immune disorder, electroconvulsive therapy remains a significant therapy for treating this condition. Thirteen healthy subjects formed the control group. The Positive and Negative Syndrome Scale (PANSS) total score was determined before and after the electroconvulsive therapy. The concentration of IL-10 increased in TRS patients, whereas the level of IL-5 decreased. Changes in the PANSS total score were found before and after the electroconvulsive therapy. The results for the evaluation of cytokine levels in the patients with therapy-resistant schizophrenia and those treated with electroconvulsive therapy indicated a reduced PANSS total score and a balance of pro- and anti-inflammatory cytokines following electroconvulsive therapy [17].

The rehabilitation of patients with schizophrenia depends on their cognitive capabilities. In this context, a group of Japan scientists under the leadership of Ayumi Yamanushi examined fifteen patients with schizophrenia, who underwent cognitive training using the computer program RehaCom^®^ (a computer-assisted cognitive remediation therapy) and whose cognitive abilities were compared with patients who did not receive this cognitive training [18]. Patients received this cognitive remediation therapy twice weekly over twelve weeks. In fact, the cognitive abilities and PANSS total scores were improved in those patients who received the cognitive training with RehaCom^®^. Functional level, intrinsic motivation, and cognition improved in this group. Accordingly, the RehaCom^®^ program improved cognition and other outcomes in patients with schizophrenia [18]. In the largest psychiatric hospital in Romania, scientists under the leadership of Floris Petru Iliuta examined the therapeutic effects of electroconvulsive therapies (ECT) in individuals with schizophrenia and recurrent depression [19]. The authors studied pharmacological treatment, effectiveness, and clinical and socio-demographic profiles in patients (249 subjects) who received ECT during the last ten years [19]. The most frequent diagnosis for which ECT was carried out was recurrent depression followed by schizophrenia [19]. In total, 111 patients required re-hospitalization after performing ECT, whereas 138 did not require hospital readmission [19]. Moreover, differences in the number of ECT sessions required, diagnosis, socio-demographic data and psychotropic medication were observed between both previous groups [19]. Males (25–44 years; 7–12 ECT sessions required) with schizophrenia needed re-hospitalization after ECT treatment, but those patients not requiring re-hospitalization were mostly females (45–65 years; 4–6 ECT sessions required) with recurrent depression [19]. Neuropsychiatrists in Romania under the leadership of Cristian Petrescu examined neurological soft signs in eighty-one patients with schizophrenia and performed a neurological examination one and six months after the first examination [20]. The neurological soft signs (NSS)—which included motor coordination, sensory integration, and sequencing of complex motor acts—correlated with the state of the disease and were independent from the adverse effects promoted by neuroleptics. In fact, a limited impact on NSS was reported by neuroleptics [20]. NSS occurred above all after administration of the first-generation antipsychotic drug, haloperidol. A correlation between negative symptoms of schizophrenia and NSS was also reported [20]. NSS were often seen in patients with schizophrenia who showed a severe course of the disorder [20]. Thus, it seems that NSS is related to schizophrenic symptoms and not extrapyramidal symptoms commonly treated by neuroleptics. Finally, an Italian group of researchers under the leadership of Lorenzo Moccia undertook a literature review about the possibility of improving negative symptoms of schizophrenia by administering marketed drugs [21]. Whereas positive schizophrenia symptoms are well treated by the available antipsychotic drugs, negative schizophrenia symptoms cannot be emended by the current treatment. The authors discuss all possible drugs that could alleviate negative schizophrenia symptoms, including antipsychotic, antidepressant and anti-inflammatory drugs as follows: minocycline, citalopram, reboxetine, escitalopram, mirtazapine, duloxetine, selegiline, desmopressin, memantine, L-carnitine, *N*-acetylcysteine, citicoline, riluzole, cilostazol, simvastatin, sildenafil, granisetron, tropisetron, cerebrolysin, sarsasapogenin, pioglitazone, palmitoylethanolamide, pentoxifylline, sulforaphane and saffron. The antipsychotic drugs that best improved negative schizophrenia symptoms were olanzapine and cariprazine [21].

Overall, the scholars who contributed to this Special Issue have demonstrated that risperidone ISM^®^ achieved sustained and rapid therapeutic plasma levels in schizophrenia [15]; that schizophrenia patients have independent risk factors for secondary diseases from arteriosclerosis [16]; that electroconvulsive therapy decreased PANSS total score and favored a balance of pro- and anti-inflammatory cytokines [17]; that the computer program RehaCom^®^ improved cognitive capabilities [18], and that NSS is related to schizophrenic symptoms and not the extrapyramidal symptoms commonly treated by neuroleptics [20]. Moreover, the therapeutic effects of electroconvulsive therapies (ECT) in schizophrenia [19] and the pharmacological interventions to improve the negative symptoms in this disease were reported [21]. This Special Issue provides researchers with measures to improve negative schizophrenia symptoms and cognition, which cannot be ameliorated by current pharmacotherapies [19,21]. It is important to highlight that patients with schizophrenia should be supported to improve prognoses and reduce the risk for secondary diseases, such as coronary heart disease [16]. In the future, long-term examinations of new antipsychotic drugs and accompanying therapies that are not pharmacological—for example, psychoeducation—should be investigated in a large group of patients with schizophrenia, with the aim of improving the prognosis of psychotic disorders. The findings reported in this Special Issue increase our knowledge of schizophrenia from different perspectives, explore new therapeutic strategies, and open new directions of research.
